# Probing the Role of Nascent Helicity in p27 Function as a Cell Cycle Regulator

**DOI:** 10.1371/journal.pone.0047177

**Published:** 2012-10-12

**Authors:** Steve Otieno, Richard Kriwacki

**Affiliations:** 1 Department of Structural Biology, St. Jude Children's Research Hospital, Memphis, Tennessee, United States of America; 2 Department of Microbiology, Immunology and Biochemistry, The University of Tennessee Health Science Center, Memphis, Tennessee, United States of America; Weizmann Institute of Science, Israel

## Abstract

p27 regulates the activity of Cdk complexes which are the principal governors of phase transitions during cell division. Members of the p27 family of proteins, which also includes p21 and p57, are called the Cip/Kip cyclin-dependent kinase regulators (CKRs). Interestingly, the Cip/Kip CKRs play critical roles in cell cycle regulation by being intrinsically unstructured, a characteristic contrary to the classical structure-function paradigm. They exhibit nascent helicity which has been localized to a segment referred to as sub-domain LH. The nascent helicity of this sub-domain is conserved and we hypothesize that it is an important determinant of their functional properties. To test this hypothesis, we successfully designed and prepared p27 variants in which domain LH was either more or less helical with respect to the wild-type protein. Thermal denaturation experiments showed that the ternary complexes of the p27 variants bound to Cdk2/Cyclin A were less stable compared to the wild-type complex. Isothermal titration calorimetry experiments showed a decrease in the enthalpy of binding for all the mutants with respect to p27. The free energies of binding varied within a much narrower range. *In vitro* Cdk2 inhibition assays showed that the p27 variants exhibited disparate inhibitory potencies. Furthermore, when over-expressed in NIH 3T3 mouse fibroblast cells, the less helical p27 variants were less effective in causing cell cycle arrest relative to the wild-type p27. Our results indicate that the nascent helicity of sub-domain LH plays a key role mediating the biological function of p27.

## Introduction

Strict temporal control of cell cycle phase transitions is governed primarily by a family of highly conserved serine/threonine kinases termed the Cyclin-dependent kinases (Cdks) [1], [2]. Given their cardinal role in controlling faithful duplication of cells, Cdk activity is tightly regulated through post-translational modifications and direct protein-protein interactions. Cdks require the binding of a regulatory subunit referred to as a cyclin (forming Cdk/cyclin complexes), [3]–[5] and phosphorylation on a conserved threonine by a Cdk activating kinase (CAK) [6]–[8] for complete activation. In addition, Cdk’s and Cdk/cyclin complexes interact with additional regulatory proteins referred to as the Cyclin-dependent kinase regulators (CKRs). There are two families of CKRs. First, the Ink4 family [that includes p15^Ink4b^ (p15), [9] p16^Ink4a^ (p16), [10] p18^Ink4c^ (p18), [11] and p19^Ink4d^ (p19) [11]] inhibits Cdk activity by binding to monomeric Cdk4 and Cdk6 and preventing formation of complexes with D-type cyclins [12]. Second, the Cip/Kip proteins [that includes p21^Waf1/Cip1/Sdi1/Cap20^ (p21), [13]–[15] p27^Kip1^ (p27), [16]–[18] and p57^Kip2^ (p57) [19], [20]] bind to and inhibit fully activated Cdk2/cyclin A and Cdk2/cyclin E resulting in cell cycle arrest at the G_1_ to S transition [21]. Interestingly, p21 and p27 have been reported to form catalytically active ternary complexes upon interacting with Cdk4/cyclin D and Cdk6/cyclin D [21], [22]. Recent studies from our lab have uncovered a mechanism involving tyrosine phosphorylation of p21 and p27 that explains the paradoxical outcome of their interaction with Cdk4/cyclin D and Cdk6/cyclin D [23]–[25].

An N-terminal segment of the Cip/Kip proteins termed the kinase inhibitory domain (KID) has been shown to be necessary and sufficient for inhibition of Cdk/cyclin complexes [17]. The KID is modular and is comprised of a cyclin-binding sub-domain (termed D1), a 22 residue sub-domain (termed LH) that joins D1 to a Cdk binding sub-domain (termed D2) [26]. Before binding Cdk complexes, the Cip/Kip proteins are largely disordered and devoid of stable tertiary structure; proteins with these features are generally termed intrinsically disordered proteins (IDPs) [27]. The Cip/Kip proteins were amongst the first IDPs to be shown to fold extensively upon binding their biological targets [28], [26]. The structure of p27-KID bound to Cdk2/cyclin A from crystallography illustrated the end state after folding upon binding and provided insight into the molecular basis for Cdk inhibition by the Cip/Kip proteins [29]. In this complex (PDB accession code 1JSU), the D1 sub-domain of p27 adopts an extended conformation upon binding the cyclin A subunit of the complex, sub-domain LH folds into an amphipathic α-helix, and sub-domain D2 folds into amphipathic β-hairpin, a β-strand, and a 3_10_ helix on binding Cdk2. However, it is important to note that even though they are largely disordered prior to binding their targets, the Cip/Kip proteins have been shown in isolation to exhibit partially populated secondary structures within some segments; these nascent structures have been termed intrinsically folded structural units (IFSUs) [30].

We previously reported that the most prominent IFSU within the p27-KID is sub-domain LH which exhibits nascent α-helical characteristics before binding to Cdk/cyclin complexes [30]. The nascent helical features of sub-domain LH are conserved amongst the three Cip/Kip proteins [20], [26], [28]; interestingly, however, its amino acid sequence is not, with only three out of the 22 residues are conserved as identities. As explained above, the LH sub-domain folds into an amphipathic α-helix when p27 binds Cdk/cyclin complexes. The folding of p27 upon binding these complexes is accompanied by a large and unfavorable entropic cost. We hypothesized that the partial α-helical character of sub-domain LH observed before the Cip/Kip proteins bind Cdk/cyclin complexes reduces the entropic cost associated with folding upon binding and that perturbation of this conserved structural feature would affect (i) the thermodynamics of their interactions with Cdk/cyclin complexes and (ii) their capacity to inhibit Cdk catalytic activity.

In the current study, we have tested this hypothesis by preparing p27-KID variants with either a more or less helical LH sub-domain and studying their interactions with Cdk2/Cyclin A. We also prepared a third p27-KID variant in which the LH sub-domain was replaced with a loop which lacked a sequence relation to the Cip/Kip proteins. The structure of the mutants was analyzed by circular dichroism spectropolarimetry (CD) and the thermodynamics of their interaction with Cdk2/Cyclin A with isothermal titration calorimetry (ITC). CD was also used to determine the thermal stability of the ternary complexes formed by the mutants on binding Cdk2/Cyclin A. In addition, we determined the Cdk2/Cyclin A inhibition potencies of the p27-KID variants by performing *in vitro* kinase inhibition assays. The capacity of the mutants to regulate the G_1_/S phase transition was investigated by performing cell cycle arrest assays with NIH 3T3 mouse fibroblast cells transduced with retroviruses encoding the p27 variants. Together, the results of these experiments provide novel insights into the role of nascent helicity within the LH sub-domain in mediating the kinase inhibitory function of p27.

## Results

### Secondary Structure Analysis

**Figure 1:**
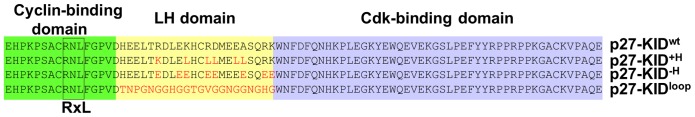
Sequences of p27-KID LH sub-domain variants used in this study. The mutated residues within sub-domain LH are colored red. RxL motif residues are boxed.

**Figure 2:**
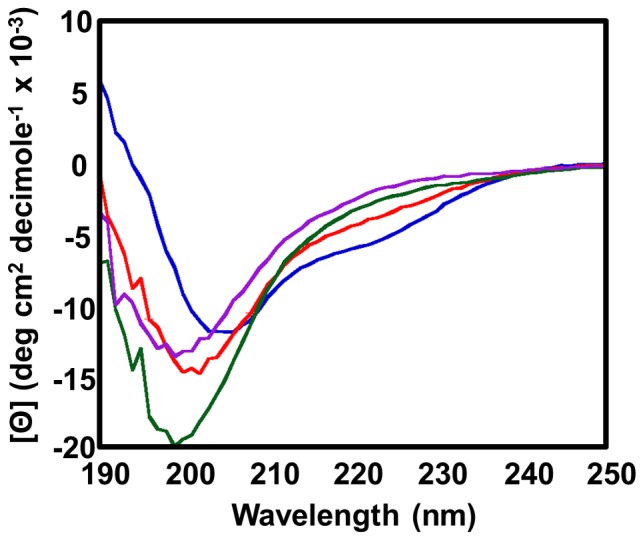
Comparative CD spectra of the p27-KID LH sub-domain variants. Each sample contained 20 µM of protein in a buffer containing 20 mM sodium phosphate pH 7.0, and 1 mM DTT. The spectra for p27-KID^wt^, p27-KID^+H^, p27-KID^−H^, and p27-KID^loop^ are colored red, blue, green, and purple, respectively.

We designed (as detailed in File S1), expressed, and purified a number of p27-KID variants in order to obtain mutants that were more or less helical than the wild-type KID construct (p27-KID^wt^, p27-KID^+H^ and p27-KID^−H^, respectively), and a mutant exhibiting random coil secondary structure in the LH sub-domain (p27-KID^loop^). We determined the secondary structure of the soluble proteins by CD and selected three constructs for our studies (Figure 1). The secondary structure of the selected mutants was analyzed using CD (Figure 2). The values of ellipticity at 222 nm (Θ_222_) for the p27-KID^wt^, p27-KID^+H^, p27-KID^−H^, and p27-KID^loop^ were −4.0, −5.8, −3.0, and −2.1(the units in all cases are degree cm^2^ decimole×10^−3^), respectively. p27-KID^+H^ and p27-KID^−H^ were more and less helical than p27-KID^wt^, respectively. The p27-KID^loop^ variant exhibited features of a complete random coil, with little ellipticity at 222 nm and a minimum at 200 nm. We previously showed that the weak helical ellipticity at 222 nm for p27-KID^wt^ could be attributed to the LH sub-domain [26]. In that study, we demonstrated that D1 and D2 were largely unstructured and that the nascent helical character of p27-KID could be ascribed to the LH Domain. Therefore, the variation in ellipticity at 222 nm for the p27-KID variants can be attributed to the differences in the helical contents caused by the mutations. In summary, our CD analysis established that our designed mutants had the desired LH domain helical contents and were suitable for the designed studies.

### Thermodynamics of the Interaction of the p27-KID Variants with Cdk2/cyclin A

**Figure 3:**
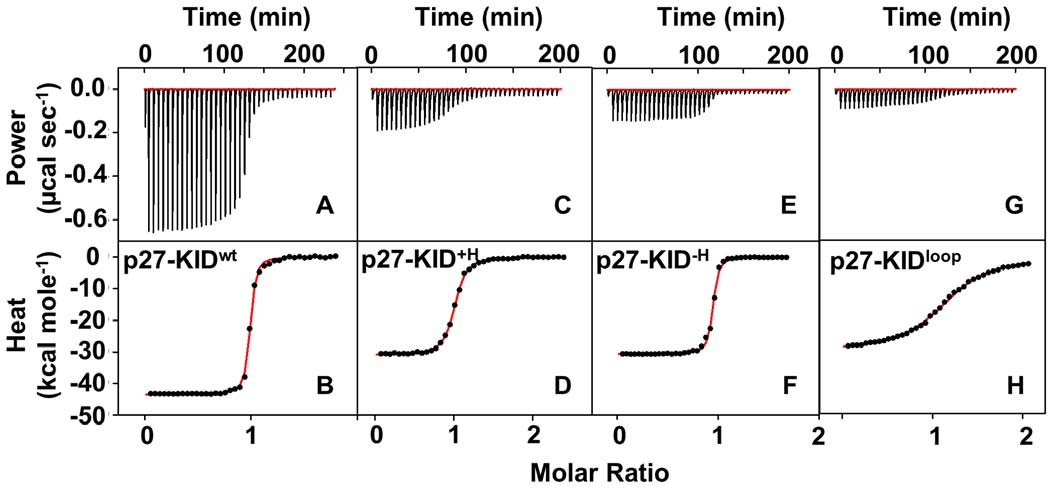
Isothermal titration calorimetry. Raw titration calorimetry data and binding isotherms, respectively, for (a, b) p27-KID^wt^, (**c, d**) p27-KID^+H^, (**e, f**) p27-KID^−H^, and (**g, h**) p27-KID^loop^ binding to Cdk2/cyclin A.

p27-KID^wt^ folds extensively on binding the Cdk2/cyclin A complex [26]. The interactions of the linker helix variants with the Cdk2/cyclin A complex, like those of p27-KID^wt^, involve dramatic folding coupled with binding and, consequently, were accompanied by large entropic costs (manifested as positive values of the entropy term, −TΔS; Table 1). The –TΔS values observed for p27-KID^wt^, p27-KID^+H^, p27-KID^−H^, and p27-KID^loop^ were +32.2±1.2 Kcal mol^−1^, +22.3±2.5 Kcal mol^−1^, +19.0±1.0 Kcal mol^−1^, and +20.0±1.4 Kcal mol^−1^, respectively. The folding of the mutants upon binding to the Cdk2/cyclin A complex resulted in a large number of favorable contacts and consequently release of a large quantity of heat when they bound to the Cdk2/cyclin A complex. Nevertheless, the enthalpies of interaction for the variants were smaller in magnitude relative to that for binding of p27-KID^wt^. The enthalpies of interaction (ΔH) for p27-KID^wt^, p27-KID^+H^, p27-KID^−H^, and p27-KID^loop^ were −43.8±1.4 Kcal mol^−1^, −32.7±2.6 Kcal mol^−1^, −30.5±0.8 Kcal mol^−1^, and −29.1±1.2 Kcal mol^−1^, respectively (Figure 3). However, these results suggest strongly that none of the mutants folded to the same extent as p27-KID^wt^.

These enthalpies of interaction, for the wild-type p27-KID and the variants, exceeded the respective unfavorable interaction entropies and resulted in overall favorable Gibbs free energies of interaction (ΔG). ΔG values of −11.6±0.3 Kcal mol^−1^, −10.4±0.2 Kcal mol^−1^, −11.5±0.1 Kcal mol^−1^, and −9.4±0.2 Kcal mol^−1^ were obtained for p27-KID^wt^, p27-KID^+H^, p27-KID^−H^, and p27-KID^loop^, respectively. The ΔΔG value for p27-KID^+H^ with respect to that of p27-KID^wt^ was +1.2 Kcal mol^−1^ [ΔΔG  =  ΔG (p27-KID^+H^)−ΔG (p27-KID^wt^)], demonstrating that increasing the helical content of the linker domain did not make the interaction of p27-KID with the Cdk2/cyclin A complex more favorable; in fact, it actually caused it to be slightly less favorable. The reduced helical content of p27-KID^−H^ did not affect the ΔG of interaction with Cdk2/cyclin A based on the finding that the ΔG values for p27-KID^wt^ and p27-KID^−H^ were identical within error. The thermodynamics of the interaction of the p27-KID^loop^ mutant with Cdk2/cyclin A, highlights the significance of the nascent helicity of p27-KID. Replacing the native LH sub-domain with a loop composed of residues with a low helical propensity made the interaction with Cdk2/cyclin A less favorable than with the wild-type sub-domain; the ΔΔG value for p27-KID^loop^ with respect to p27-KID^wt^ was +2.2 Kcal mol^−1^. As previously reported [26], the folding of the LH sub-domain is a key step as p27 sequentially folds upon binding to Cdk2/cyclin A. The ITC results for p27-KID^loop^ binding to Cdk2/cyclin A show that disruption of the nascent helicity of the LH sub-domain is thermodynamically unfavorable. In summary, the results of our thermodynamic studies show that perturbation of the helical features of the LH sub-domain affected the thermodynamics of the interaction of p27-KID with the Cdk2/cyclin A complex.

**Table 1:** Thermodynamic binding parameters of the p27-KID mutants.

Interaction	*K*_d_ (nM)	ΔG^a^ (kcal mol^−1^)	ΔH (kcal mol^−1^)	-TΔS^b^ (kcal mol^−1^)
p27-KID^wt^ + Cdk2/cyclin A	3.3±1.6	−11.6±0.3^c^	−43.8±14	+32.2±1.2
p27-KID^+H^ + Cdk2/cyclin A	23.4±6.1	−10.4±0.2	−32.7±2.6	+22.3±2.5
p27-KID^−H^ + Cdk2/cyclin A	3.7±0.9	−11.5±0.1^c^	−30.5±0.8	+19.0±1.0
p27-KID^loop^ + Cdk2/cyclin A	138.3±43.1	−9.4±0.2	−29.1±1.2	+19.7±1.4

In all the experiments above, the stoichiometry of binding (n) was determined to be one (1), with experimental error within 10%. The errors reported above are the standard deviations from the mean of three independent experiments.

a Calculated with the equation ΔG = −RT ln(1/*K*_d_ ).

b Calculated with the equation −TΔS  =  ΔG−ΔH.

c The c values (c  =  (*K*_d_)^−1^ * [Cdk2/cyclin A]) for these p27-KID variants were >500 and the ΔG values reported here are taken as estimates of the upper limits of *K*_d_. Ideal c values for accurate determination of *K*_d_, have a range of 1 to 500. Specifically, the c values for the binding of p27-KID^wt^ and p27-KID^−H^ to Cdk2/cyclin A complex are 2,212 and 1,698, respectively.

### Thermal Stability of the p27-KID/Cdk2/cyclin A Ternary Complexes

**Figure 4:**
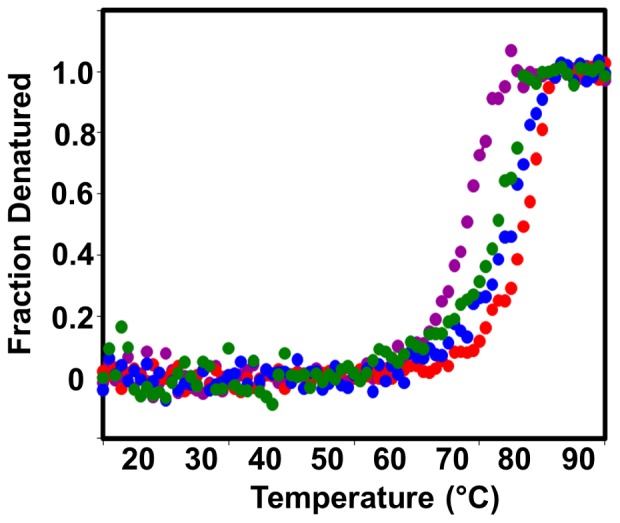
Thermal stability of complexes. Thermal denaturation curves of ternary complexes formed by the interaction of Cdk2/cyclin A and p27-KID^wt^ (red), p27-KID^+H^ (blue), p27-KID^−H^ (green), or p27-KID^loop^ (purple).

The Cdk2/cyclin A complex exhibits modest thermal stability, with an apparent melting temperature (T_m_^app^) of 54.7±0.6°C [31]. When p27-KID^wt^ binds to the Cdk2/cyclin A complex, the thermal stability of the ternary complex formed was greatly increased to 82.3±0.5°C. The T_m_^app^ values for the p27-KID^+H^, p27-KID^−H^, and p27-KID^loop^ ternary complexes were 80.3±0.2°C, 78.5±0.6°C, and 73.0±0.3°C respectively (Table 2, Figure 4). These results indicated that perturbation of the helical content of the linker domain decreased the thermal stability of the ternary complexes. Reducing the helical content (p27-KID^−H^) or completely abolishing the nascent helical character (p27-KID^loop^) had a larger effect on thermal stability when compared to increasing the helical content (p27-KID^+H^). The observation of destabilization of the ternary complex formed by the p27-KID^+H^ variant suggested that the flexibility of the linker domain may play a significant role in the folding of the p27-KID^wt^ on binding to the Cdk2/cyclin A complex to form a thermally stable ternary complex. On the other hand, the T_m_^app^ values obtained for the p27-KID^−H^ and p27-KID^loop^ mutants suggest that excessive flexibility of the linker domain is also destabilizing.

### Cdk2/cyclin A Inhibitory Potencies of the p27-KID Variants

It has previously been shown that p27-KID is a potent inhibitor of the kinase activity of the Cdk2/cyclin A complex [17]. Related studies performed in our laboratory showed that a peptide corresponding to the Cdk binding domain of p27 (Figure 1) could not completely inhibit kinase activity [32]. This peptide inhibited Cdk2/cyclin A kinase activity with an IC_50_ of about 125 nM but was associated with 25% residual kinase activity even at peptide concentrations as high as 17 µM (data not shown). These data suggested that the Cdk binding domain and other portions of the KID (the cyclin binding and LH sub-domains) are required for complete and potent Cdk inhibition. However, the specific role of the linker domain in the mechanism of Cdk2/cyclin A inhibition by the KID is not understood.

**Figure 5:**
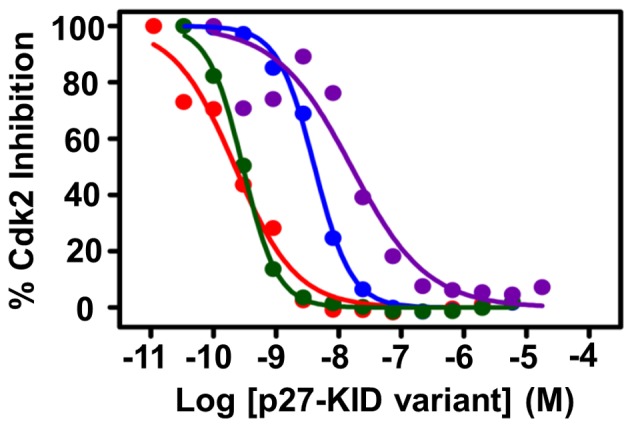
Cdk2/cyclin A inhibition curves for the p27-KID variants. The curves for p27-KID^wt^, p27-KID^+H^, p27-KID^−H^, and p27-KID^loop^ are colored red, blue, green, and purple, respectively.

For kinase inhibition assays, a very low concentration of the Cdk2/cyclin A kinase complex (80 pM per reaction) was used to prevent inhibitor depletion which would otherwise result in IC_50_ values that were greater than the true values. All of the sub-domain LH variants inhibited kinase activity in the low nanomolar range. However, none of the variants were more potent than the wild-type KID. The IC_50_ values for p27-KID^wt^, p27-KID^+H^, p27-KID^−H^, and p27-KID^loop^ were 220 pM, 4.1 nM, 290 pM, and 16.0 nM, respectively (Table 3, Figure 5, Figure S1). The results obtained for the p27-KID^loop^ variant are very interesting because they showed that a KID molecule formed by coupling the cyclin and Cdk binding domains via a linker of random sequence could inhibit the Cdk2/cyclin A complex with an IC_50_ as low as 16 nM. This inhibitory potency, however, is reduced more than 50-fold relative to that of p27-KID^wt^. Furthermore, increasing the helical content of sub-domain LH decreased the inhibition potency of the p27-KID. For example, the p27-KID^+H^ variant was >17-fold less potent than the wild-type KID. However, reducing the helical content relative to wild-type (p27-KID^−H^) did not diminish the inhibition potency of the KID; we observed that the p27-KID^−H^ variant was almost as potent as the wild-type KID. In summary, while increasing the helical content of sub-domain LH reduced the Cdk2/cyclin A inhibition potency of p27-KID, decreasing the helical content did not have a significant effect. In contrast, abolishing the nascent helicity of sub-domain LH (p27-KID^loop^) greatly reduced the inhibition potency.

**Table 2:** Apparent thermal denaturation temperatures (T_m_^app^) of the ternary complexes formed by the p27-KID mutants on binding the Cdk2/cyclin A complex.

Complex	T_m_^app^ (°C)
p27-KID^wt^/Cdk2/cyclin A	82.3±0.5
p27-KID^+H^/Cdk2/cyclin A	80.3±0.2
p27-KID^−H^/Cdk2/cyclin A	78.5±0.6
p27-KID^loop^/Cdk2/cyclin A	73.0±0.3

### Cell Cycle Arrest Capacity of the Sub-domain LH Variants

**Figure 6:**
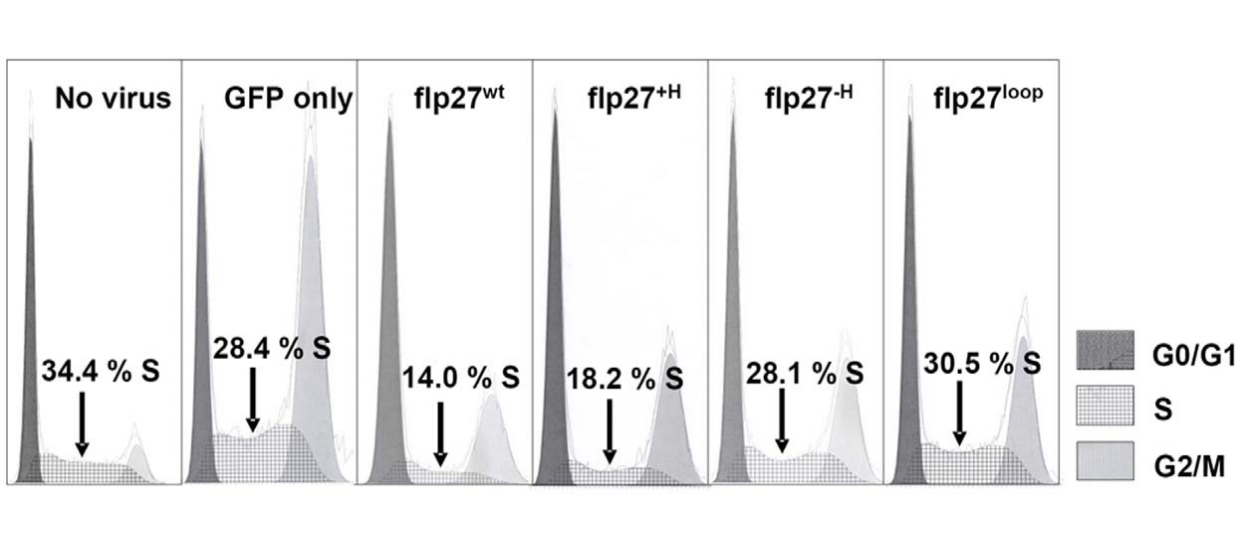
Cell cycle arrest analysis of full-length p27 LH sub-domain variants. The percentage of cells observed in S phase is indicated.

Polyak, *et. al,* discovered p27 as a potent inhibitor of Cdk2 complexes during the G_1_ phase of the cell cycle [16]. To investigate the consequences of perturbation of sub-domain LH on p27-dependent cell cycle arrest, we prepared retroviruses encoding full-length versions of the p27 variants (e.g. also including the C-terminal domain which plays a role in regulation of p27 stability) [27], [33]. These constructs are referred to as p27^wt^, p27^+H^, p27^−H^, and p27^loop^. The retroviruses were used to transduce NIH 3T3 mouse fibroblast cells and the fraction of cells in S phase was used as a measure of cell cycle arrest at the G_1_ to S transition (Table 4, Figure 6). The retroviruses co-expressed GFP as a marker of transduction efficiency. As expected, p27^wt^ arrested the cells with only 14±1.0% of the cells observed in S phase relative to 28.4±2.4% in those transduced only with GFP. Increasing the helical content of the LH sub-domain did not have a significant impact on the cell cycle arrest capacity as 18.2±1.7% of cells transduced with p27^+H^ were observed in the S phase. Helical destabilization of the LH sub-domain completely abrogated the ability to cause cell cycle arrest with 28.1±1.1% (which is within the error range of the GFP control) of cells observed in S phase for the p27^−H^ mutant. The cell cycle effects of replacing the LH sub-domain with a loop (p27^loop^) were similar to those observed due to destabilizing the LH sub-domain (p27^−H^), with 30.5±1.2% of cells observed in S phase for the p27^loop^ variant. The cell cycle analysis of the capacity of the p27 variants to cause cell cycle arrest showed that reduction or elimination of the nascent helicity of sub-domain LH had dramatic effects on the cell cycle arrest capacity of p27; however, increasing the helical content did not have a significant effect.

**Table 3:** Inhibition potencies of the p27-KID mutants.

Variant	IC_50_ (nM)	95% confidence intervals of IC_50_ (nM)^a^
p27-KID^wt^	0.22	0.17–0.28
p27-KID^+H^	4.1	3.3–5.1
p27-KID^−H^	0.29	0.26–0.33
p27-KID^loop^	16.0	9.9–25.7

a Error is reported as the 95% confidence interval of the IC_50_ from a curve fit of a triplicate data set.

## Discussion

While primary structure is not conserved, the nascent helical secondary structure of the LH sub-domain is conserved amongst members of the Cip/Kip Cdk inhibitor family [32]. We probed the consequences of perturbing this conserved structural element on the biophysical and functional properties of p27 by studying the interactions of p27 variants, in which the helical content of the linker domain was either increased (p27-KID^+H^), decreased (p27-KID^−H^), or completely eliminated (p27-KID^loop^), with Cdk2/cyclin A. The original rationale for these experiments was the past observation (demonstrated again here, Table 1) that the folding and binding of p27-KID to Cdk2/cyclin A is associated with a large and unfavorable entropy change (e.g., large and positive value of –TΔS). Based on this, we hypothesized that a p27-KID construct with enhanced helical character within the LH sub-domain would exhibit a reduced entropic penalty and therefore experience increased affinity for Cdk2/cyclin A. However, our results indicated that factors in addition to entropy came into play when p27-KID and the p27-KID variants bound to Cdk2/cyclin A.

**Table 4:** Cell cycle analysis of NIH 3T3 mouse fibroblast cells expressing the p27 LH sub-domain constructs.

Cell cycle phase	No plasmid	Empty vector (GFP only)	p27^wt^	p27^+H^	p27^−H^	p27^loop^
**G_0_/G_1_**	55.3±2.6	22.2±2.1	57.9±0.4	48.5±3.0	38.7±3.2	39.9±2.3
**S**	34.4±2.1	28.4±2.4	14.0±1.0	18.2±1.7	28.1±1.1	30.5±1.2
**G_2_/M**	10.3±1.3	49.5±2.6	28.2±0.6	33.3±1.4	33.1±3.0	29.6±1.5

The values represent the percentage of cells at the various phases of the cell cycle. Triplicate experiments were done for each control and p27 variant.

At first, it seemed as though the binding entropy data for p27-KID^+H^ supported our initial hypothesis. For example, the value of −TΔS values for p27-KID^+H^ binding to Cdk2/cyclin A (+22.3±2.5 kcal mol^−1^) corresponded to a reduced binding-associated entropy change in comparison with that observed for p27-KID^wt^ (+32.2±1.2 kcal mol^−1^). The value of –TΔΔS for this pair of reactions was −9.9 kcal mol^−1^ [−TΔΔS = [−TΔS (p27-KID^+H^)] – [−TΔS (p27-KID^wt^)]]. However, examination of other thermodynamic parameters associated with this binding reaction indicated that the favorable enthalpy of binding was reduced by an amount that was slightly greater in absolute value than the reduction in entropic penalty [ΔΔH  =  ΔH (p27-KID^+H^)−ΔH (p27-KID^wt^) = +11.1 kcal mol^−1^]. This approximate enthalpy/entropy compensation probably arose from incomplete folding of the variant LH sub-domain of the p27-KID^+H^ construct upon binding to Cdk2/cyclin A, as was observed previously for another series of LH sub-domain variants [32]. The phenomenon of reduced enthalpy/entropy compensation was observed for the other p27 LH sub-domain variants examined here (Table 1). In this earlier study, the conclusion that reduced enthalpy/entropy compensation, as observed here for all of the LH sub-domain variants, was due to incomplete LH sub-domain folding upon binding was verified through NMR studies showing that the cyclin- and Cdk-binding sub-domains of the p27 constructs folded similarly upon binding to Cdk2/cyclin A (ruling out contributions from differences in the folding and binding of these sub-domains to the observed thermodynamic differences). Interestingly, the enthalpy and entropy values for the p27-KID^−H^ variant gave rise to an overall ΔG value that was essentially identical to that observed for p27-KID^wt^. This result indicates that nascent helicity within the LH sub-domain and extensive folding upon binding of this sub-domain are not requirements for tight binding to Cdk2/cyclin A. However, the highly disordered features of the p27-KID^loop^ variant were associated with less favorable binding to Cdk2/cyclin A than for the other constructs, suggesting that intrinsic features of the LH sub-domain of the p27-KID^−H^ variant, apart from its heightened disorder, are favorable for binding. Results from another recent study by Wang, et al., [34] indicate that the surface of Cdk2/cyclin A that is spanned by the LH sub-domain is electro-positive (see Suppl. Fig. 4 in ref. [34]), possibly giving rise to favorable electrostatic interactions with negatively charged residues within the wild-type and variant LH sub-domains. The LH sub-domain of p27-KID^−H^ is enriched in glutamic acid residues while that of p27-KID^loop^ is depleted in these, possibly partly explaining the differences observed in their respective thermodynamic binding parameters. In summary, modulating the nascent helicity of the LH sub-domain of p27 did not give rise to the changes in binding energy (ΔG) predicted based on a simple model considering only the change in entropy of this sub-domain upon binding to Cdk2/cyclin A. Our findings suggest that features of the natural p27 LH sub-domain that were associated with maximal binding enthalpy and presumably maximal folding upon binding were not captured in the variant LH sub-domain constructs. In contrast, however, two of these variants did exhibit relatively tight binding to Cdk2/cyclin A (p27-KID^+H^ and p27-KID^−H^; *K*_d_ increased <10-fold in comparison with wild-type), indicating that extensive folding upon binding (within the LH sub-domain for these constructs) is not a requirement for disordered protein/folded protein interactions. This latter observation is consistent with recent observations on persistent disorder within the dynamic complex formed by Sic1 and Cdc4 [35].

Other *in vitro* assays showed that the p27-KID LH sub-domain variants exhibited rank order behavior similar to that observed for ΔG values determined using ITC. For example, while all p27 LH sub-domain variants caused stabilization, p27-KID^wt^ and p27-KID^−H^ stabilized their complexes with Cdk2/cyclin A to the greatest extent and the p27-KID^loop^ variant to the smallest extent. This rank order behavior was recapitulated in the results from the *in vitro* Cdk2 inhibition assays. In these assays, p27-KID^wt^ and p27-KID^−H^ were the most potent inhibitors of Cdk2/cyclin A, with p27-KID^+H^ and p27-KID^loop^>10-fold and >50-fold less potent, respectively, than the former two constructs. Collectively, our ITC, thermal stabilization and *in vitro* biochemical results support the view that alteration of the LH sub-domain within the kinase inhibitory domain of p27 significantly influences the thermodynamic and functional properties of the full KIDs.

The biological consequences of LH sub-domain alteration were probed by determining the cell cycle arrest properties of the corresponding full-length p27 variants (Table 4). The p27 constructs with the wild-type (p27^wt^) and more helical (p27^+H^) LH sub-domains were equally potent in causing arrest in G_1_ phase of the cell division cycle. The result for p27^+H^ is in contrast to the reduced activity of the corresponding KID construct in the *in vitro* assays. The two other full-length LH sub-domain variants, p27^−H^ and p27^loop^, caused essentially no reduction in the fraction of cells in S phase but were associated with an increased G_0_/G_1_ content (relative to the GFP only control), indicative of partial cell cycle arrest. These results may reflect differential interactions of the various p27 LH sub-domain variants with the repertoire of Cdk/cyclin complexes which control entry into and progression of the cell division cycle from G_0_ to G_1_ and then to S phase. Alteration of the ability of the related Cdk inhibitor, p21, to promiscuously interact with and inhibit multiple Cdk/cyclin complexes (through lengthening or shortening of the LH sub-domain) was previously shown to alter the ability to cause complete cell cycle arrest [34]. It is possible that the p27^−H^ and p27^loop^ LH sub-domain variants exhibit altered Cdk/cyclin binding promiscuity, which is manifested as incomplete cell cycle arrest.

We observed a dependence of the ability of the KID of the Cip/Kip proteins to interact with a Cdk/cyclin complex on the features of the so-called “linker” or LH sub-domain even though it has been reported that this sub-domain does not significantly contribute to the favorable thermodynamics of interactions with Cdk2/cyclin A [26]. For example, the binding of a peptide corresponding to the wild-type p21 LH sub-domain to Cdk2/cyclin A could not be detected using ITC [34]. These observations highlight the mechanistic complexity of the functional interplay between different segments (or sub-domains) of a disordered protein domain. For example, substitution of the natural LH sub-domain with a glycine-rich linker led to a >50-fold decrease in Cdk2 inhibitory potency. While we have gained further insight into “disorder/function relationships” for the p27 kinase inhibitory domain, our results point out the challenges associated with detailed characterization of dynamic protein complexes [36]. Disorder within complexes has significant entropic ramifications which can significantly influence the energetic landscape which controls binding kinetics and affinity. However, disorder within complexes is challenging to characterize in terms of ensembles of individual molecular states as well as the populations of these states and the time scale of exchange between them. Even more challenging is to relate this type of “molecular picture” of a dynamic complex to thermodynamic parameters. The present study contributes data on relationships between thermodynamic binding parameters and the corresponding functional parameters for closely related disordered protein domains. Future studies will seek to provide the currently lacking molecular picture so as to correlate structure and dynamics with thermodynamics and function for this class of prototypical disordered proteins.

## Materials and Methods

### Design of p27-KID Mutants

Our approach of designing p27-KID mutants with linker domains that were either more or less helical than the wild-type p27-KID was to substitute solvent exposed residues within the linker domain with residues that are known to either stabilize or destabilize helices. Mutating the solvent exposed residues minimizes disruption of contacts between p27 and the Cdk2/cyclin A complex. This reduced the possibility that the results obtained from the succeeding studies were a consequence of the disruption of contacts between p27 and Cdk2/cyclin A. The solvent accessible surface area (SASA) for each residue within the linker domain was analyzed using the GETAREA program [37]. The input used for the program was the pdb file of the p27-KID/Cdk2/cyclin A complex (1JSU). Residues which had more than 50% of their total surface area exposed to solvent were selected as suitable candidates for mutagenesis. From the output, 10 residues had more than 50% of their surface areas exposed (Table S1). The 10 candidate residues were further analyzed using the Insight II program (Accelrys, San Diego, CA). The PDB file, 1JSU, was used as an input and all the atoms within a 6 Å radius of every atom of each candidate residue were analyzed. This was done to determine whether any of the candidate residues interacted with the residues of the binary complex. No close contacts were observed for the 10 candidate residues (Table S1). After this analysis, all the 10 candidate residues were determined to be suitable for mutagenesis. Design approaches for specific mutants are detailed in File S1. A list of the designed mutants are detailed in Table S2.

### Preparation of Proteins

p27-KID^wt^ protein was expressed and purified as previously described [26]. The p27-KID variants were ligated into a pET-28a plasmid (Novagen, Gibbstown, NJ) either as a 6x-His fusion protein construct (p27-KID^+H^ ) or GST-6x-His fusion constructs (p27-KID^−H^ and p27-KID^loop^) and expressed in *Escherichia coli BL21* (DE3) cells (Novagen, Gibbstown, NJ ). The proteins were purified by nickel affinity chromatography and then digested with thrombin to remove the fusion tags. This was followed by boiling for 20 minutes and separation of the supernatant (purified protein) and the pellet by centrifugation. Full length human Cdk2 phosphorylated at threonine 160 (Cdk2) and truncated human cyclin A (residues 173–432) were expressed and purified using established protocols [31] [26]. The Cdk2/cyclin A kinase complexes were made by mixing purified cyclin A and purified Cdk2 for 30 minutes at 4°C. The binary complex was then purified by size exclusion chromatography (S75 resin, GE Healthcare, Piscataway, NJ). The ternary complexes were prepared by mixing either p27-KID^wt^ or the LH sub-domain variants with purified Cdk2/cyclin A complex in a 1.1∶1.0 ratio, incubating the mixture for 30 minutes and purifying the complexes by gel filtration chromatography (S200 resin, GE Healthcare, Piscataway, NJ). Prior to the ITC experiments or ternary complex preparation, p27-KID^wt^ and its variants were treated with 100 mM DTT at room temperature for 30 minutes followed by gel filtration chromatography purification. Protein concentrations were determined by UV absorbance at 280 nm as detailed in File S1.

### Isothermal Titration Calorimetry (ITC)

ITC experiments were done in a buffer composed of 20 mM HEPES pH 7.5, 300 mM NaCl, and 5 mM DTT, at 25°C using a VP-ITC calorimeter (GE Healthcare, Piscataway, NJ). p27-KID^wt^ or its LH variants (in the syringe) were titrated into Cdk2/cyclin A complex (in the cell). The titration sequence included a single 2 µL injection followed by 39 injections, 6 µL each, with a spacing of 300 seconds between the injections. The protein concentrations were as follows: 75 µM p27-KID^wt^ into 7.4 µM Cdk2/cyclin A; 56 µM p27-KID^+H^ into 4.2 µM Cdk2/cyclin A; 42 µM p27-KID^−H^ into 4.4 µM Cdk2/cyclin A; and 36.4 µM p27-KID^loop^ into 3.1 µM Cdk2/cyclin A. OriginLab software (GE Healthcare, Piscataway, NJ).) was used to fit the raw data to a 1∶1 binding model.

### Denaturation Studies

The thermal stability of the ternary complexes formed when p27-KID^wt^ or its variants interacted with Cdk2/cyclin A was determined using an AVIV model 62A DS circular dichroism spectropolarimeter equipped with a thermoelectric temperature controller (AVIV, Lakewood, NJ). Each ternary complex sample contained 400 nM of protein in 1 mM sodium phosphate buffer pH 7, 25 mM NaCl and 1 mM DTT. Denaturation was monitored by ellipticity at 222 nm in the temperature range of 15°C to 95°C. Ellipticity readings were taken every 1°C at a heating rate of 5°C min^−1^ in a 1 cm path length quartz cuvette with constant stirring. Each experiment was performed in triplicate. The curves were fit as previously described [31] to determine the values of the apparent thermal denaturation temperature (T_m_^app^). Apparent thermal denaturation temperatures are reported because the denaturation process is irreversible due to protein precipitation after denaturation.

### Kinase Activity Assays

Cdk2/cyclin A kinase (80 pM) was mixed with 2.5 µM histone H1 (Upstate) and varying quantities of p27-KID^wt^ or its mutants and incubated at 4°C for 60 minutes. This was followed by addition of 6 µCi γ ^32^P-ATP (PerkinElmer, Inc) to each reaction and incubation at 30°C for 35 minutes. Each reaction had a total volume of 15 µL. The sample buffer contained 20 mM HEPES pH 7.3, 25 mM sodium β-glycerolphosphate, 15 mM MgCl_2_, 16 mM EGTA, 0.5 mM Na_3_VO_4_ and 10 mM DTT. The reactions were stopped by adding SDS-PAGE gel loading buffer and were then analyzed by SDS-PAGE. A phosphoimager (GE Healthcare, Piscataway, NJ) was used to quantify the bands on the gel and data fitting was done using the Graphpad Prism software (Graphpad Software, Inc., San Diego, CA). Each curve was fit to a triplicate data set to derive values of p27-KID (or p27-KID variant) concentration that caused 50% inhibition (IC_50_ values) of the kinase complex and the errors are reported as the 95% confidence interval.

### Preparation of Retroviral Packaging Vector DNA

The coding sequences for full-length versions of human p27 containing either the wild-type LH sub-domain or the variants, additionally with a C-terminal HA tag, were sub-cloned into an MSCV packaging vector and these vectors were used for preparation of packaged viruses that expressed HA-tagged wild-type and the LH sub-domain variants of full-length p27 and GFP [38].

### Preparation of Viruses

Retroviruses encoding HA tagged full-length constructs of the p27 variants were packaged in 293T cells. 100 mm×10 mm plates of 293T cells were cultured at 37°C in Dulbecco’s Modified Eagle Medium (DMEM) supplemented with 10% fetal bovine serum (FBS), 2% of 200 mM glutamine (vol:vol), and 1% of 10,000 µg/µl penicillin/streptomycin (vol:vol). Hereafter, the DMEM supplements are referred to as additives. 3×10^6^ cells were cultured on each plate. After 24 hours, the cell culture media (with additives) was substituted with additive free media (DMEM only) and incubated for 3 hours. During this time, transfection cocktails were prepared.

The cocktails contained a single helper DNA (containing the viral gag-pol-env genes), shuttle vector DNA plasmid, fugene 6 transfection reagent (Roche, Basel, Switzerland) and additive free DMEM media. The helper DNA was obtained from Dr. Martine Roussel (St. Jude Children’s Research Hospital, Memphis, TN). In every cocktail, the ratio of the volume of the fugene reagent to the amount of the shuttle vector (by mass) was varied to identify the ratio that yielded the highest viral titre. The volume of the fugene reagent was kept constant at 15 µl and the amount of the shuttle vector added was varied to give different fugene to shuttle vector ratios. Cocktails with volume of fugene to mass of shuttle plasmid DNA ratios of 1∶20, 1∶15, 1∶12, and 1∶10 were prepared for each construct (each construct had 4 cocktails). A constant 6 µg of helper DNA was added to each coctktail. Serum free media was added to bring the total volume of each cocktail to 700 µl. Before addition of fugene to the cocktails, it was first warmed to room temperature according to manufacturer’s instructions. For each cocktail, the reagents were added in the following order: (i) serum free media, (ii) helper DNA, (iii) vector DNA, and (iv) fugene. The cocktails were then mixed. After 3 hours, the transfection cocktails were added to the incubated cells.

After 20 hours the additive free media was replaced with 10 ml of fresh media supplemented with additives. In the following steps, all the media was supplemented with additives unless stated otherwise. After 6 hours, the media was aspirated from the plates and 5 ml of media was added to each plate. After 18 hours, 5 ml of the media containing virus was harvested and 5 ml of fresh media was added to each plate. 8 hours later, 5 ml of media containing virus was again harvested and a fresh 7 ml of fresh media added to each plate.

Virus-containing media (7 ml) was harvested a final time after an additional 20 hours. The harvested virus was filtered using a 0.22 µm steriflip filter (Millipore, Billerica, MA) and stored at 4°C.

### Viral Transduction and Cell Cycle Arrest Analysis

3×10^5^ NIH 3T3 mouse embryo fibroblast cells were cultured on 100×10 mm plates in DMEM media with additives (additives specified in section 2.9 above). After 20 hours, the media was aspirated from each plate. 5 ml of cold virus (virus kept in ice) supplemented with 30 µl of 1 mg/ml of polybrene (6 µl of polybrene for every ml of virus) was added to each plate and incubated at 37°C. After 3 hours, 10 ml of DMEM media with additives was added to each plate. After 48 hours, the cells were washed, re-suspended in additive-free DMEM media, and the level of protein expression determined using GFP fluorescence analysis (Flow Cytometry Facility, St. Jude Children’s Research Hospital, Memphis, TN). Before GFP analysis, viable cells were separated from non-viable cells by propidium iodide (PI) staining. For PI staining, the cells were first mixed until a single suspension of cells was realized. The cells were then counted and washed once with phosphate buffered saline (PBS). The supernatant was decanted and the cells were resuspended in PI solution (0.05 mg/ml propidium iodide) at 1×10^6^ cells/ml. This was followed by treatment with RNAse for 30 minutes at room temperature. Prior to and after the RNAse step, the cells were kept in ice. The cells were then filtered using a 40 µm-diameter mesh (Small Parts Inc., Miami Lakes, FL) to eliminate large multicellular clumps that might clog the flow cytometer. The filtered cells were analyzed by flow cytometry.

For cell cycle analysis, viral solutions with high viral titres, as determined by GFP analysis, were used to transduce NIH 3T3 cells as described above. The cells were washed, and analyzed for viral transduction using GFP analysis. The top 10% GFP positive cells were stained by PI and analyzed for DNA content. Each cell cycle arrest analysis was performed in triplicate for each construct.

## Supporting Information

Figure S1 Raw data for Cdk2/cyclin A inhibition assays. Phosphorimager analysis of SDS-PAGE results of phosphorylation of Histone H1 (PO_4_-H1) by Cdk2/cyclin A in the presence of increasing concentrations of p27-KID^wt^ or the LH sub-domain variants.
(TIF)

Table S1 Solvent exposure of the residues in the linker domain of p27.
(DOCX)

Table S2 Linker domain sequences for the p27-KID variants designed to be more and less helical.
(DOCX)

File S1 (DOCX)
