# Supplementary figures and images for: Probing the Role of Nascent Helicity in p27 Function as a Cell Cycle Regulator
Source: PLoS One. 2012 Oct 12;7(10):e47177. doi: 10.1371/journal.pone.0047177 (PMC3470550; doi:10.1371/journal.pone.0047177)

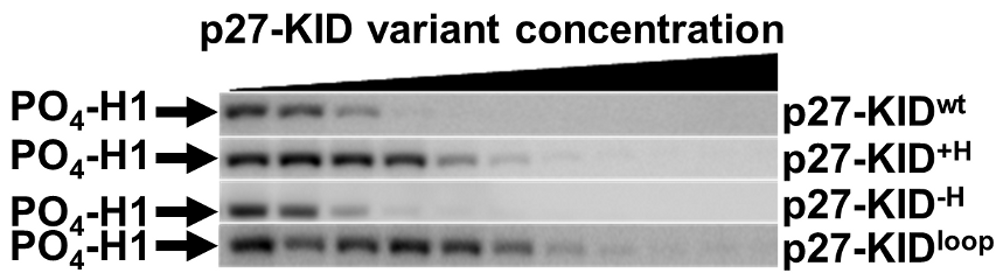

Supplement: Figure S1 — Raw data for Cdk2/cyclin A inhibition assays. Phosphorimager analysis of SDS-PAGE results of phosphorylation of Histone H1 (PO4-H1) by Cdk2/cyclin A in the presence of increasing concentrations of p27-KIDwt or the LH sub-domain variants. (TIF) [file pone.0047177.s001.tif]
