# Supplementary material for: Probing the Role of Nascent Helicity in p27 Function as a Cell Cycle Regulator
Source: PLoS One. 2012 Oct 12;7(10):e47177. doi: 10.1371/journal.pone.0047177 (PMC3470550; doi:10.1371/journal.pone.0047177)
Supplement: Table S1 — Solvent exposure of the residues in the linker domain of p27. (DOCX) [file pone.0047177.s002.docx]

Table S1. Solvent exposure of the residues in the linker domain of p27.

| **Residue** | **Percentage of surface area exposed to solvent** |
| --- | --- |
| His38 | 40.9 |
| **Glu39** | **84.1** |
| **Glu40** | **79.9** |
| Leu41 | 1.9 |
| Thr42 | 22.7 |
| **Arg43** | **56.7** |
| Asp44 | 33 |
| Leu45 | 0.4 |
| Glu46 | 26.9 |
| **Lys47** | **76.2** |
| His48 | 27.2 |
| Cys49 | 13.2 |
| **Arg50** | **67.1** |
| **Asp51** | **55.4** |
| Met52 | 20.2 |
| Glu53 | 37.9 |
| **Glu54** | **81.6** |
| **Ala55** | **87.1** |
| Ser56 | 14.6 |
| Gln57 | 30 |
| **Arg58** | **81.3** |
| **Lys59** | **56.1** |

The residues selected for mutagenesis are in bold font**.**
