# Supplementary material for: Probing the Role of Nascent Helicity in p27 Function as a Cell Cycle Regulator
Source: PLoS One. 2012 Oct 12;7(10):e47177. doi: 10.1371/journal.pone.0047177 (PMC3470550; doi:10.1371/journal.pone.0047177)
Supplement: Table S2 — Linker domain sequences for the p27-KID variants designed to be more and less helical. (DOCX) [file pone.0047177.s003.docx]

**Table S2. Linker domain sequences for the p27-KID variants designed to be more and less helical.**

| **Variant** | **Sequence of linker domain** |
| --- | --- |
| *p27-KID^wt^* | *H****EE****LT****R****DLE****K****HC****RD****ME****EA****SQ****RK****^a^* |
| p27-KID^A10^ | HAALTADLEAHCAAMEAASQAA^b^ |
| p27-KID^A22^ | AAAAAAAAAAAAAAAAAAAAAA^b^ |
| p27-KID^SL1^ | HLELTLDLELHCLLMELLSQRK^b^ |
| p27-KID^SL2^ | HEELTLDLELHCLLMELLSQRK^b^ |
| *p27-KID^SL3^ (p27-KID^+H^)* | *HEELTKDLELHCLLMELLSQRK^b^* |
| p27-KID^SL4^ | HEELTKDLELHCKLMEELSQKK^b^ |
| p27-KID^SL5^ | HLELTLDLEEHCLKMELESQLK^b^ |
| p27-KID^SL6^ | HLLLTLDLELHCLLMELASQLL^b^ |
| *p27-KID^-H^* | *HEELTEDLEEHCEEMEEESQEE^b^* |
| *p27-KID^loop^* | *TNPGNGGHGGTGVGGNGGNGHG^b^* |

Sequences used in the present study are italicized.

^a^ The residues determined to be suitable for mutagenesis are underlined and are in bold font in the linker domain sequence of the wild-type p27-KID (p27-KID^wt^).

^b^ The mutated residues are underlined.
