# Supplementary material for: Probing the Role of Nascent Helicity in p27 Function as a Cell Cycle Regulator
Source: PLoS One. 2012 Oct 12;7(10):e47177. doi: 10.1371/journal.pone.0047177 (PMC3470550; doi:10.1371/journal.pone.0047177)
Supplement: File S1 — (DOCX) [file pone.0047177.s004.docx]

**SUPPORTING INFORMATION**

**for**

**Probing the Role of Nascent Helicity in p27 Function as a Cell Cycle Regulator**

Steve Otieno^1^, and Richard Kriwacki ^1,2*^

^1^Department of Structural Biology, St. Jude Children's Research Hospital, Memphis, TN 38105

^2^Department of Microbiology, Immunology and Biochemistry, The University of Tennessee Health Science Center, Memphis, TN 38163

**Materials and Methods**

*Design of p27-KID mutants*

Two different strategies were used in the design of mutants with stabilized linker domains. In the first approach, alanine was used as a possible stabilizer of the helical structure of the linker domain. Two alanine mutants were designed. In the first alanine mutant, all the residues that were selected for mutagenesis were substituted with alanine residues. Alanine was chosen because previous studies have shown that alanine residues stabilize and have a high propensity to occur in α-helices.[[1-3](#_ENREF_1)] This mutant, in which the 10 candidate residues were substituted with alanines, was designated p27-KID^A10^. In the second alanine mutant, all the 22 residues in the linker domain were substituted with alanines. This mutant was designated p27-KID^A22^. This approach did not yield a suitable mutant for our studies. In the second approach, leucine was used as a potential stabilizer of the linker domain helix. Like alanine, leucine has also been shown to stabilize and have a high propensity to occur in α-helices.[[4](#_ENREF_4)]^,^[[2](#_ENREF_2)] Hydrophobic interactions between leucine side chains on one face of an α-helix shield the backbone amides and carboxyl groups from hydrogen bonding with water, thus stabilizing the helical conformation.[[5](#_ENREF_5)] One disadvantage of leucine, however, is its hydrophobic nature. To counter the hydrophobicity of leucine, we designed a library of mutants in which the number of leucines replacing the candidate residues was balanced with charged, polar residues. The substituted residues in these mutants consisted of either 7 leucines and 3 polar residues, 6 leucines and 4 polar residues, 5 leucines and 5 polar residues, etc. We also ensured that the polar residues had a high helical propensity. The leucines were strategically positioned three and four residues apart in the helix. Past studies have shown that leucines positioned three and four residues apart stabilize α-helices.[[6](#_ENREF_6)] In addition, leucines positioned four residues apart is one of the most commonly observed pair-wise side chain interactions in protein α-helices.[[7](#_ENREF_7)] A total of 6 mutants were designed. The mutants were designated p27-KID^SL1^ (SL1; stabilized by leucine, construct 1), p27-KID^SL2^, p27-KID^SL3^, p27-KID^SL4^, p27-KID^SL5^, and p27-KID^SL6^. In the p27-KID^SL6^ mutant, all the candidate residues were substituted with leucines. This mutant was designed to test if charged residues that were determined to be unsuitable for mutagenesis could counter the hydrophobicity of the 10 leucines. Of the leucine mutants, only the p27-KID^SL3^ (designated p27-KID^+H^ in this study) was both soluble and more helical than p27-KID^wt^.

For the less helical mutant, the candidate residues were replaced with glutamic acid (Glu) residues. The premise behind choosing Glu residues is that electrostatic repulsion between the negatively charged Glu side-chains should destabilize the helix backbone.[[8](#_ENREF_8)] In addition, the substituting residues close to the carboxy terminus of the helix with Glu residues would result in further destabilization of the helix due to repulsion of the negatively charged Glu side-chain and the negative carboxy-terminus helix dipole.[[9](#_ENREF_9)] Another advantage of using glutamate is that the polar Glu side-chain should enhance the solubility of this mutant. This mutant was designated p27-KID^-H^.

A mutant with a flexible loop comprised of mainly glycine residues, a known helix breaker, and other residues with a low propensity for being in α-helices was also designed. The residues that comprised this mutant were chosen with an objective of minimizing helical secondary structure in the LH sub-domain. The number of residues in the loop equaled 22, as in the p27 linker domain. This mutant was designated p27-KID^loop^. Table S2 shows the linker domain sequences of all the mutants designed.

*Determination of Protein Concentrations*

We used UV absorbance at 280 nm to determine the concentrations of the proteins used in this study. The absorbance readings were taken with the proteins in a denaturing buffer containing 20 mM phosphate pH 6.5, and 6.0 M guanidinium hydrochloride [[10](#_ENREF_10),[11](#_ENREF_11)]. The molar extinction coefficients were determined using the ProtParam tool on the ExPASY server (<http://us.expasy.org/tools/protparam.html>). The extinction coefficient for p27-KID^wt^, p27-KID^+H^, p27-KID^-H^ and p27-KID^loop^ was determined to be 15,220 M^-1^ cm^-1^. Cdk2, cyclin A, and the Cdk2/cyclin A complex had extinction coefficients of 35,560 M^-1^ cm^-1^, 31,860 M^-1^ cm^-1^, and 33,710 M^-1^ cm^-1^, respectively. The concentrations of the ternary complexes of the p27-KID^wt^ and its variants were determined using an extinction coefficient of 24,465 M^-1^ cm^-1^.

References

1. Pace CN, Scholtz JM (1998) A helix propensity scale based on experimental studies of peptides and proteins. Biophys J 75: 422-427.

2. Chou PY, Fasman GD (1974) Conformational parameters for amino acids in helical, (-sheet, and random coil regions calculated from proteins. Biochemistry 13: 211-221.

3. Lopez-Llano J, Campos LA, Sancho J (2006) Alpha-helix stabilization by alanine relative to glycine: roles of polar and apolar solvent exposures and of backbone entropy. Proteins 64: 769-778.

4. Chakrabartty A, Kortemme T, Baldwin RL (1994) Helix propensities of the amino acids measured in alanine-based peptides without helix-stabilizing side-chain interactions. Protein Sci 3: 843-852.

5. Luo P, Baldwin RL (2002) Origin of the different strengths of the (i,i+4) and (i,i+3) leucine pair interactions in helices. Biophys Chem 96: 103-108.

6. Luo P, Baldwin RL (2002) Origin of the different strengths of the (i,i+4) and (i,i+3) leucine pair interactions in helices. Biophys Chem 96: 103-108.

7. Klingler TM, Brutlag DL (1994) Discovering structural correlations in alpha-helices. Protein Sci 3: 1847-1857.

8. Moser R (1992) Design, synthesis and structure of an amphipathic peptide with pH-inducible haemolytic activity. Protein Eng 5: 323-331.

9. Shoemaker KR, Kim PS, Brems DN, Marqusee S, York EJ, et al. (1985) Nature of the charged-group effect on the stability of the C-peptide helix. Proc Natl Acad Sci U S A 82: 2349-2353.

10. Gill SC, von Hippel PH (1989) Calculation of protein extinction coefficients from amino acid sequence data. Anal Biochem 182: 319-326.

11. Gasteiger E, Hoogland C, Gattiker A, Duvaud S, Wilkins MR, et al. (2005) Protein Identification and Analysis Tools on the ExPASy Server. In: Walker JM, editor. Proteomics Protocols Handbook. Totowa, NJ Humana Press.
